# Echocardiographic detection of intracardiac thrombus complicating ventriculoatrial shunt

**DOI:** 10.4103/0974-2069.52815

**Published:** 2009

**Authors:** Neeraj Awasthy, S Radhakrishnan, Savitri Shrivastava

**Affiliations:** Department of Congenital and Pediatric Heart Disease, Escorts Heart Institute and Research Center (EHIRC), New Delhi, India

## CLINICAL SUMMARY

A two-year-old girl, a known case of congenital hydrocephalus, had a ventriculoperitoneal shunt, which was converted to a ventriculoatrial (VA) shunt, due to peritonitis. Following this procedure, the child was admitted with fever to the pediatric ward of a general hospital. She was referred to us for echocardiography, to rule out endocarditis. Blood counts of the child were within normal limits and repeated blood culture, including a fungal culture, was negative. Echo evaluation showed a dense hyperechoic mass measuring 9 × 7 mm. It was attached to the atrial end of the ventriculoatrial shunt and was highly mobile [Figure 1a and b]. This was diagnostic of a thrombus at the site of the VA shunt. The thrombus was going across the tricuspid valve into the right ventricle during diastole [Figure 2]. The child was started on heparin and subsequently underwent thrombolysis with streptokinase. Steptokinase Injection was used at 2000 units per kg stat (through the shunt) followed by infusion of 1000 units per kg per hour with partial thromboplastin time (PTT) monitoring. A follow-up echo done 18 days later showed a significant reduction in the size of the thrombus and measured 2.5 × 3 mm. As the mass had not completely resolved, the patient was started on oral anticoagulants (Acitrom) and is under regular follow up.

**Figure 1(a):**
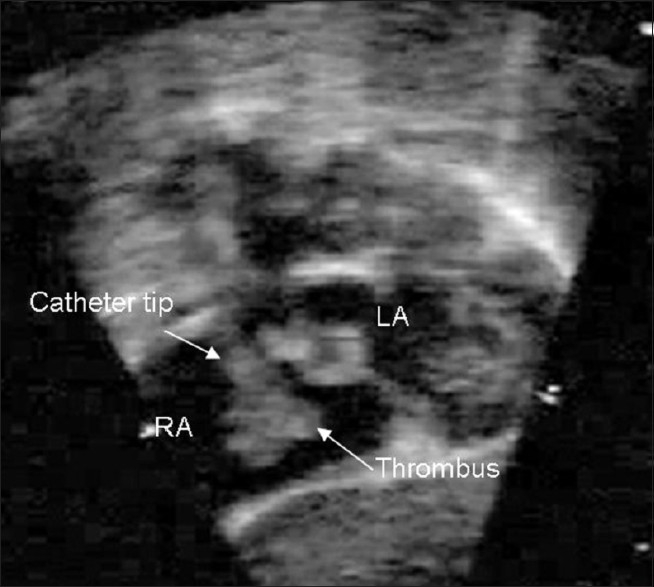
Two-dimensional echocardiography, a subcostal saggital view showing thrombus attached to the catheter tip in the right atrium (RA)

**Figure 1(b):**
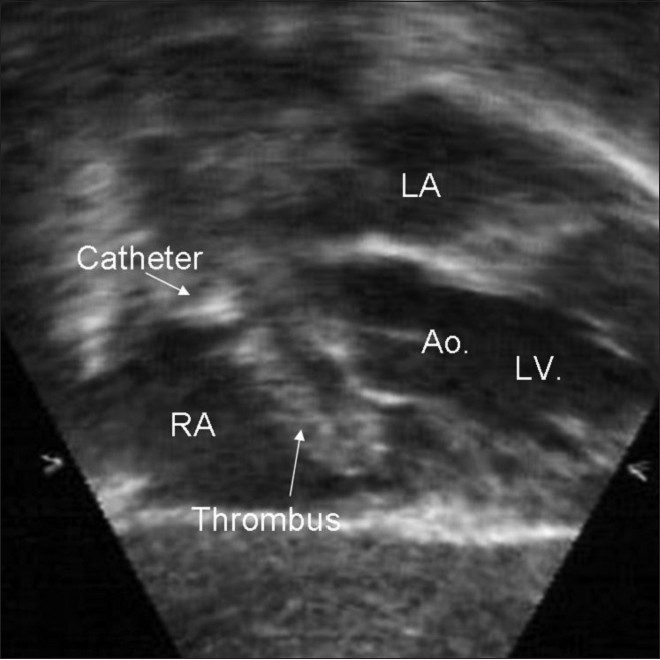
Two-dimensional echocardiography, a modified subcostal saggital view showing thrombus attached to the catheter tip in the right atrium (RA)

**Figure 2:**
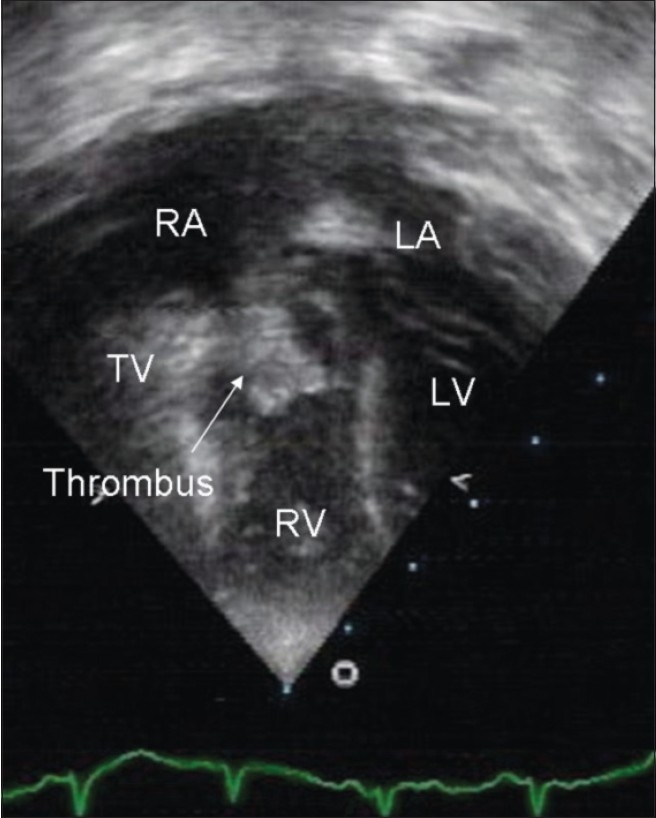
Two-dimensional echocardiography, a four chamber view showing thrombus going through the tricuspid valve in diastole LA: Left atrium, LV: Left ventricle, Ao.: Aorta, RA: Right atrium, RV: Right ventricle

## DISCUSSION

While evaluating a child with a VA shunt, one should carefully look for a thrombus at the atrial end. Although uncommon, it has important prognostic and therapeutic implications. Such a thrombus can repeatedly embolize in pulmonary circulation resulting in the development of pulmonary hypertension and cor pulmonale.[1–3] A large thrombotic mass embolizing in the lung can cause life-threatening complications.[2,4] Some others can present as shunt dysfunctions.[5] These signs are often subtle, and when present in a patient with a VA shunt, must raise the suspicion of a thrombus at the distal end of the catheter. The incidence of clinically significant pulmonary emboli is approximately 3.2% in all patients with VA shunts and carries a high mortality of 50-100%, thus necessitating early diagnosis and treatment.[5]

Echocardiography is the procedure of choice, to rule out the presence of an intracardiac thrombus related to the intracardiac end of the VA catheter. The intracardiac thrombus can either be free floating in the cardiac chambers or, more often than not, is attached to the distal end of the catheter, as in our case. It can also be partially or completely attached to the tricuspid valve. Echo is also useful in following up these children with respect to migration, progression, and resolution of the thrombus.

Given the frequency and severity of complications associated with the VA shunt, it is recommended that the patients be followed up twice a year, with at least one echocardiographic evaluation each year.[5] It is advisable to perform an echocardiogram before the withdrawal of the VA shunt in patients with shunt dysfunction. This can prevent a potential pulmonary thromboembolism in those with a thrombus at the atrial end of the shunt.
